# Symptomatic Vasospasm Refractory to Clazosentan after Subarachnoid Hemorrhage of Ruptured Vertebral Artery Dissecting Aneurysm: Clinical Implications from Two Contrasting Cases

**DOI:** 10.3390/medicina60091543

**Published:** 2024-09-20

**Authors:** Yasuyuki Yoshida, Tatsushi Mutoh, Junta Moroi, Tatsuya Ishikawa

**Affiliations:** 1 Department of Surgical Neurology, Research Institute for Brain and Blood Vessels, Akita Cerebrospinal and Cardiovascular Center, Akita 010-0874, Japan; 2 Department of Aging Research and Geriatric Medicine, Institute of Development, Aging and Cancer, Tohoku University, Aoba-ku, Sendai 980-8575, Japan

**Keywords:** clazosentan, cerebral vasospasm, computed tomography, subarachnoid hemorrhage, vertebral artery dissecting aneurysm

## Abstract

Clazosentan prevents vasospasms after aneurysmal subarachnoid hemorrhage (SAH). However, clinical data on patients with SAH with ruptured vertebral artery dissecting aneurysms (VADAs) are limited. We report the case of a 49-year-old male patient with mild-grade (WFNS grade 1) thick and diffuse (modified Fisher grade 3) SAH who underwent endovascular trapping of a ruptured VADA, resulting in a poor functional outcome with a modified Rankin Scale score of 4 due to severe symptomatic vasospasm refractory to clazosentan, requiring repeated rescue endovascular therapies and chronic communicating hydrocephalus. A retrospective analysis of the clot density in the basal and Sylvian cisterns, assessed by the Hounsfield unit (HU) values of serial CT scans, in this patient showed persistent higher values, distinct from another VADA case that showed a decline in HU values with a good clinical course. These results imply the limited effectiveness of clazosentan in cases of thick and diffuse SAH after a ruptured VADA, even in good-clinical-grade patients treated with less invasive modalities. The HU values may become a simple quantitative marker for predicting symptomatic vasospasms and chronic hydrocephalus.

## 1. Introduction

Subarachnoid hemorrhage (SAH), caused by the rupture of a fusiform vertebral artery dissecting aneurysm (VADA), is a well-known but rare disease representing approximately 3% of all intracranial aneurysms [1]. Although good-grade patients with ruptured VADAs usually exhibit a better clinical course, the overall patient outcomes after discharge are poor compared to patients with other saccular aneurysmal SAHs [2]. This is of particular association with the higher rebleeding rate seen with ruptured VADAs (40.5% within 24 h of initial SAH) than with other saccular aneurysms [3]. Furthermore, vasospasm or related delayed cerebral ischemia due to thick and diffuse SAH may also become an additional risk factor for worsened prognosis [4], although the prevalence of ruptured VADA is unknown.

An endothelin receptor antagonist clazosentan has demonstrated efficacy in reducing the incidence of moderate to severe angiographic vasospasm and its related delayed cerebral ischemia and morbidity [5,6]. Currently, clazosentan has become the first-line drug for the prophylactic management of vasospasms in patients with postoperative SAH in Japan [7]. According to a recent meta-analysis and systematic review, clazosentan may be regarded as a valuable adjunctive drug in managing postoperative SAH patients [8], being beneficial in a high-risk population for vasospasm with thick and diffuse SAH [9]. The amount of clinical data regarding clazosentan anti-vasospasm therapy for ruptured aneurysms in the anterior circulation has increased substantially [10,11,12,13,14,15]. However, the features of the posterior circulation, particularly in patients with a ruptured VADA, have not been reported to date. Therefore, we believe it is important to share our clinical experience of treating post-SAH symptomatic vasospasm with clazosentan following ruptured VADA.

Herein, we report a case of mild-grade SAH in a patient who successfully underwent endovascular internal trapping of the ruptured VADA, which resulted in a poor clinical outcome due to severe symptomatic vasospasm refractory to clazosentan and subsequent post-SAH hydrocephalus. In this report, we further analyzed the Hounsfield unit (HU) values of serial head computed tomography (CT) scans as a surrogate quantitative parameter of the subarachnoid clot volume and clearance for predicting vasospasms and hydrocephalus [16], and compared them with those of a contrasting case where a good clinical course was observed. This article was written in accordance with CARE (CAse REport) guidelines [17].

## 2. Case Report

### 2.1. Case Presentation

**Figure 1:**
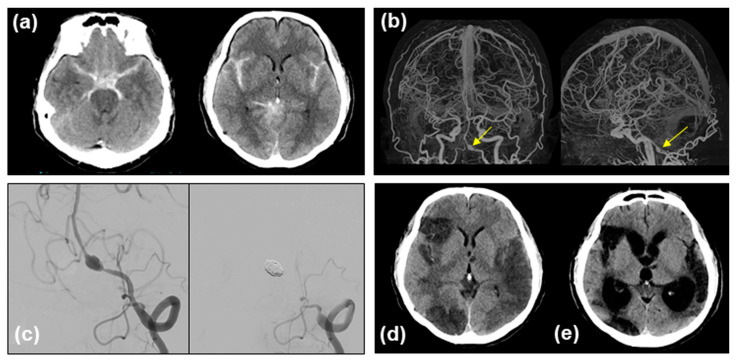
Imaging findings. Initial brain CT scan (**a**) and CT angiography (**b**). A left VADA detected by CT angiography ((**b**) arrows: (**left**), anteroposterior projection; (**right**), left lateral projection) was successfully treated by internal coil trapping. (**c**) DSA before (**left**) and after (**right**) the treatment. Follow-up CT scans on day 14 (**d**) and day 90 (**e**) suggest multiple vasospasm-related infarctions in bilateral MCA regions and the occurrence of hydrocephalus, respectively.

A 49-year-old man was brought to a tertiary hospital with the sudden onset of headache after alcohol consumption. On arrival, his level of consciousness was 15 on the Glasgow Coma Scale with no abnormal neurological findings. A CT scan of the head showed an SAH in the bilateral Sylvian fissures and basal cisterns (Figure 1a), being compatible with World Federation of Neurosurgical Societies (WFNS) clinical grade 1 (mild-grade SAH) and modified Fisher grade 3 (thick and diffuse SAH). Subsequent CT angiography of the brain revealed a left VA aneurysm (Figure 1b). The patient was then transferred to a stroke center for further treatment. A left fusiform dilating aneurysm with a “pearl and string sign” was diagnosed using digital subtraction angiography (DSA) (Figure 1c). The VADA was located at the co-dominant side and distal to the posterior internal cerebral artery. Therefore, we selected VA coil trapping as a first treatment [18], and internal trapping was successfully performed under general anesthesia (Figure 1c).

**Figure 2:**
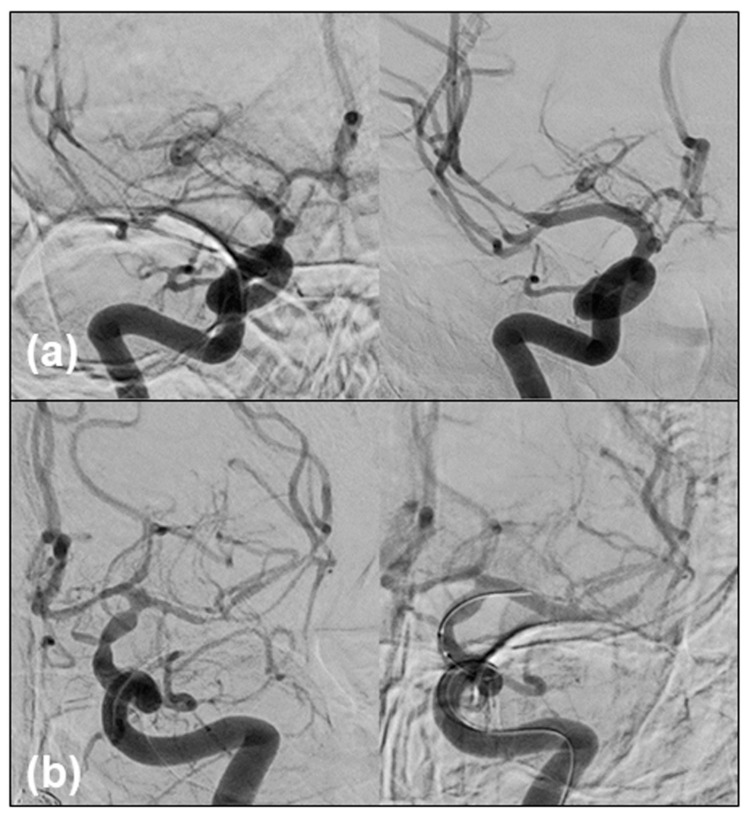
Imaging findings. (**a**) DSA of the right internal carotid artery anteroposterior projection showing vasospasm in the right MCA on day 9 (**left**), which was treated by intra-arterial fasudil and balloon angioplasty (**right**). (**b**) DSA on day 10 (**left**) demonstrated a new vasospasm in the left MCA; thus, we repeated the same endovascular therapy.

Clazosentan (10 mg/h) was initiated on day 1, and no other anti-vasospasm therapies such as oral cilostazol and/or intravenous fasudil hydrochloride [7,9,14] were used. The postoperative course was uneventful, with mild headache and low-grade fever. Lumbar drainage (30 mL) was performed on day 4, with no remarkable elevation in the cerebrospinal fluid (CSF) pressure (6 cmH_2_O). However, on day 9, the patient abruptly developed left-sided weakness and hemispatial neglect consistent with a vasospasm in the right middle cerebral artery (MCA) territory, which was confirmed by DSA and treated with intra-arterial fasudil and balloon angioplasty (Figure 2a). Institutional medical treatment for mild hypervolemia and dobutamine-induced hyperdynamic therapy [19] were ineffective. While the symptoms resolved, the patient developed aphasia the following day. Endovascular therapy was repeated for the vasospasm-affected left MCA territory (Figure 2b). 

Eventually, the patient demonstrated bilateral infarction followed by chronic communicating hydrocephalus requiring ventriculoperitoneal shunt placement (Figure 1d,e) and was transferred to a nursing home with a modified Rankin Scale score of five at 6 months.

### 2.2. Contracting Case without Vasospasm

**Figure 3:**
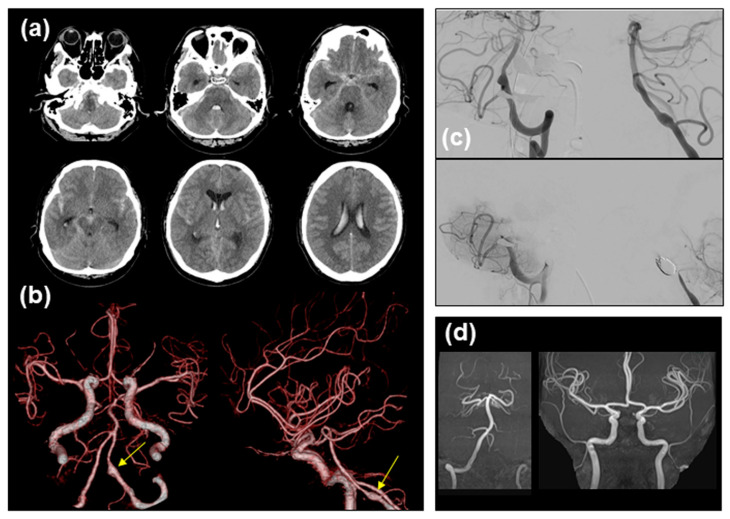
Imaging findings. Initial brain CT scan (**a**) and CT angiography ((**b**) (**left**), anteroposterior projection; (**right**), left lateral projection). A left VADA ((**b**) arrows) successfully treated by endovascular trapping. (**c**) DSA before (**upper panels**) and after (**lower panels**) the treatment. (**d**) MR angiography of the posterior (**left panel**) and anterior (**right panel**) circulation on day 14.

A 49-year-old man was brought to our hospital with a sudden onset of headache. His level of consciousness was 14 on the Glasgow Coma Scale, with no abnormal neurological findings. A head computed tomography (CT) can showed thick and diffuse SAH and intraventricular hemorrhage (Figure 3a), corresponding to WFNS grade 2 and modified Fisher grade 4). CT angiography demonstrated a left VADA located distal to the posterior internal cerebral artery (Figure 3b), which was successfully treated by endovascular VA coil trapping (Figure 3c). He received clazosentan for 14 days from day 1 after SAH onset. In contrast to the aforementioned case, his postoperative course was uneventful, without angiographic evidence of vasospasm or recanalization of the VADA (Figure 3d), and he was discharged at 1 month with a modified Rankin scale of 0.

### 2.3. Clot Density Measurements

**Figure 4:**
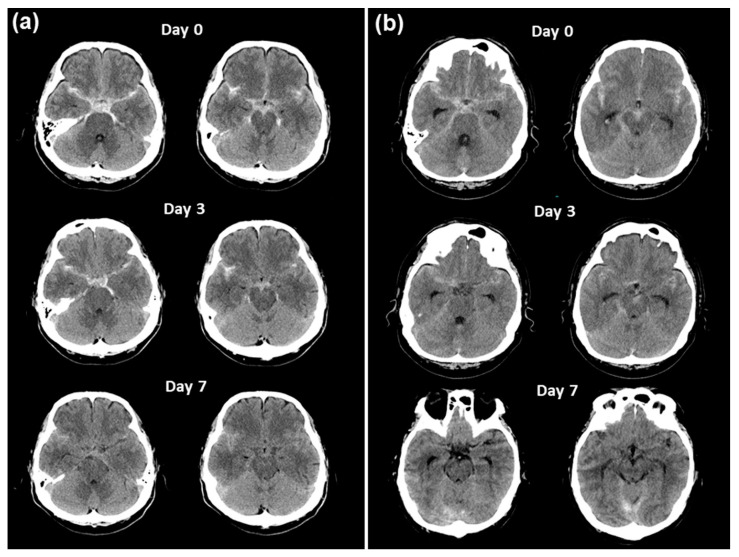
Serial brain CT imaging in the present case (**a**) with severe vasospasm on day 9 and a contracting case (**b**) with good clinical course without vasospasm following ruptured VADA.

**Figure 5:**
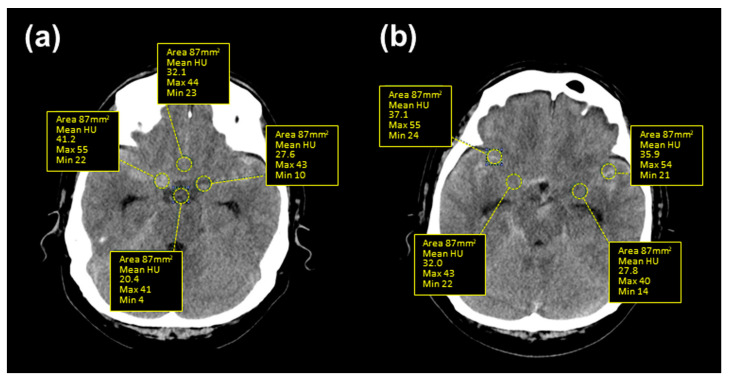
Representative brain CT imaging showing the location of analyzed HU values for assessing the density of the SAH clot. The HU values of the basal cistern (**a**) and Sylvian cistern (**b**) were measured as the average of the circular ROI values (87 mm^2^) of the bilateral carotid, interpeduncular, and lamina cisterns (**a**) and bilateral medial and lateral Sylvian cisterns (**b**), respectively. Each value has been shown to predict symptomatic vasospasm and hydrocephalus in patients with mild-grade SAH treated with endovascular coiling [16].

**Figure 6:**
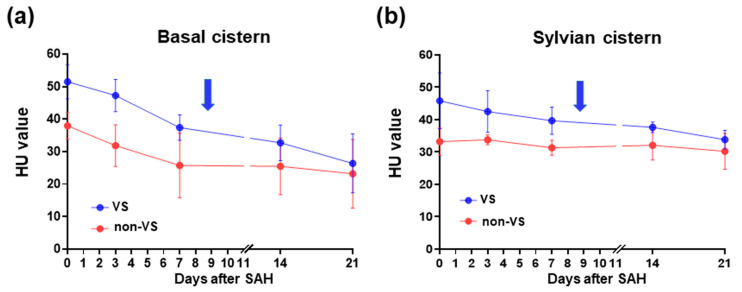
Time course changes of CT scans (**left panels**) and HU values (**right panels**) of the basal cistern (**a**) and Sylvian cisterns (**b**) in the present SAH case who developed severe vasospasm (VS) and a similar case without vasospasm (non-VS) following ruptured VADA. The bilateral cisterns’ average ROIs (expressed as means ± SD) were calculated as described (Figure 5). Note that the HU values in the present case were higher than those in the other. Arrows indicate the day when VS occurred.

In view of the brain CT imaging for both cases in the same time frame, relatively higher clot densities could be observed until the occurrence of vasospasm (Figure 4). Therefore, we retrospectively analyzed the HU values of the subarachnoid space on serial CT scans by dividing it into the basal and Sylvian cisterns (See Figure 5 for detail) because high HU values on initial CT at admission were significantly correlated with the incidence of symptomatic vasospasm (both cisterns), hydrocephalus (basal cistern), and long-term prognosis in mild-grade SAH treated with endovascular coiling [16]. The HU values for both of the basal and Sylvian cisterns (Figure 6a and 6b, respectively) in this patient remained high until the occurrence of a vasospasm, whereas the values in the other case were low at onset and declined and a good clinical course followed without vasospasm. 

## 3. Discussion

To the best of our knowledge, this is the first report of severe symptomatic vasospasm refractory to clazosentan anti-vasospasm therapy in a patient with mild-grade SAH after a ruptured VADA treated with endovascular coiling. In a recent reanalysis of the CONSCIOUS trials, the therapeutic dose of clazosentan (15 mg/h) reduced vasospasm-related morbidity at 6 weeks in a population with thick and diffuse SAH by 19% compared to a placebo (36%), with a relative risk of 0.54. However, there was still a lack of apparent positive effects on 3-month survival and clinical outcomes [20]. It should be noted that this study was conducted in patients with an aneurysmal SAH located in the anterior circulation (93%); cases of such patients with VADAs have not been reported, with only three cases located in the “distal VA”.

It is well established that extensive subarachnoid clots have prognostic significance [9]. Thus, they form the basis of the modified Fisher scale for predicting the risk of symptomatic vasospasm and delayed cerebral ischemia. In studies reporting the effects of clazosentan, an SAH clot was defined as diffuse (long axis ≥ 20 mm or present in both hemispheres; secured by surgical clipping) or thick (short axis ≥ 4 mm; secured by endovascular coiling) on the CT scan at admission. However, the impact of the location and form of the aneurysm on the clazosentan efficacy remains to be examined because similar VADA cases with thick and diffuse SAH have demonstrated good clinical outcomes without vasospasm. In a recent study, measuring the HU values provided a more objective assessment for predicting symptomatic vasospasm and hydrocephalus independent of patient-specific factors such as age or interobserver variability [16]. When we used the reported HU values in the basal cistern to predict vasospasm (cutoff value, 44.9; sensitivity, 62%; specificity, 66%; *p* = 0.001) and hydrocephalus (46 versus 40 with and without its incidence; *p* = 0.016) [16] in the present VADA-SAH case, it was determined that our patient had a higher risk for both complications than the other case with a good outcome.

In addition, the persistence of high HU values is associated with the development of vasospasms, compared to HU values that dropped rapidly. However, the clot volume and clearance rates could not be accounted for. Importantly, serial HU measurements can be performed in patients after ruptured aneurysms treated within the posterior circulation, with little or no metal artifacts (i.e., coil, stent and clip) is considered for the analysis. Considering the persistently high HU values in the subarachnoid space, more pre-emptive CSF diversion might have facilitated clot volume reduction and clearance, irrespective of the management of elevated intracranial pressure. Nevertheless, our case suggests that attention should be paid to the post-SAH clot density on admission and its serial changes, even in patients with good-grade SAH. Moreover, the higher rebleeding rate of VADA [2] may increase the incidence of thick and diffuse clot formation in the subarachnoid space, leading to severe symptomatic vasospasm refractory to clazosentan, which can abruptly worsen the patient’s condition.

Angiographically, clazosentan has shown to dilate vasospastic vessels at 24 h after starting intravenous administration in most cases, particularly in the distal arterial beds. By contrast, it achieved the reversal of large vessel vasospasm in only 27.3% of the patients [21]. Similar results have also been demonstrated in a recent Japanese single-center, observational study to compare clazosentan with a conventional protocol of oral cilostazol and intravenous fasudil hydrochloride, where clazosentan had a lower incidence of distal artery spasm (14% vs. 34%; *p* = 0.002) without significant differences in the proximal arteries (19% vs. 23%; *p* > 0.05) [7]. Indeed, there exists many biological complexities and multiple mechanisms regarding how SAH affects the brain and cerebral circulation [22]. Therefore, the limited effectiveness of clazosentan on vasospasms induced by a ruptured VADA or massive SAH clot volume should be clarified in future studies in larger populations, which may contribute to the disconnection between an effective treatment for large-artery vasospasms and clinical outcomes [23].

## 4. Conclusions

These results imply the limited effectiveness of clazosentan in cases of thick and diffuse SAH, even in good-clinical-grade patients treated with less invasive modalities. The HU value of the subarachnoid space measured by serial CT scans may become a simple quantitative marker of the clot clearance for predicting symptomatic vasospasms and chronic hydrocephalus.

## Data Availability

The datasets generated and analyzed during the present study are available from the corresponding author upon reasonable request. The data are not publicly available due to privacy and ethical restrictions.

## References

[1] Su W., Gou S., Ni S., Li G., Liu Y., Zhu S., Li X. (2011). Management of ruptured and unruptured intracranial vertebral artery dissecting aneurysms. J. Clin. Neurosci..

[2] Korai M., Kanematsu Y., Yamaguchi I., Yamaguchi T., Yamamoto Y., Yamamoto N., Miyamoto T., Shimada K., Satomi J., Hanaoka M. (2021). Subarachnoid hemorrhage due to rupture of vertebral artery dissecting aneurysms: Treatments, outcomes, and prognostic factors. World Neurosurg..

[3] Mizutani T., Aruga T., Kirino T., Miki Y., Saito I., Tsuchida T. (1995). Recurrent subarachnoid hemorrhage from untreated ruptured vertebrobasilar dissecting aneurysms. Neurosurgery.

[4] Khan S.H., Abruzzo T.A., Ringer A.J. (2009). Successful endovascular reconstruction of acutely ruptured pseudoaneurysm of the vertebral artery, complicated by isolated vertebrobasilar circulation and symptomatic vasospasm. Clin. Neurol. Neurosurg..

[5] Pontes J.P.M., Santos M.D.C., Gibram F.C., Rodrigues N.M.V., Cavalcante-Neto J.F., Barros A.D.M., Solla D.J.F. (2023). Efficacy and safety of clazosentan after aneurysmal subarachnoid hemorrhage: An updated meta-analysis. Neurosurgery.

[6] Endo H., Hagihara Y., Kimura N., Takizawa K., Niizuma K., Togo O., Tominaga T. (2022). Effects of clazosentan on cerebral vasospasm-related morbidity and all-cause mortality after aneurysmal subarachnoid hemorrhage: Two randomized phase 3 trials in Japanese patients. J. Neurosurg..

[7] Sakata H., Kanoke A., Uchida H., Haryu S., Omodaka S., Kimura N., Yoshida M., Niizuma K., Tominaga T., Endo H. (2024). Prophylactic management of cerebral vasospasm with clazosentan in real clinical practice: A single-center retrospective cohort study. Front. Neurol..

[8] Al-Salihi M.M., Saha R., Abd Elazim A., Helal A., Sabah Al-Jebur M., Al-Salihi Y., Ayyad A. (2024). The effectiveness and safety of clazosentan in treating aneurysmal subarachnoid hemorrhage: A systematic review and meta-analysis. J. Clin. Neurosci..

[9] Aldrich E.F., Higashida R., Hmissi A., Le E.J., Macdonald R.L., Marr A., Mayer S.A., Roux S., Bruder N. (2021). Thick and diffuse cisternal clot independently predicts vasospasm-related morbidity and poor outcome after aneurysmal subarachnoid hemorrhage. J. Neurosurg..

[10] Mutoh T., Aono H., Seto W., Kimoto T., Tochinai R., Moroi J., Ishikawa T. (2024). Factors Influencing Discontinuation of Clazosentan Therapy in Elderly Patients with Aneurysmal Subarachnoid Hemorrhage: A Retrospective Study from a Japanese Single Center. Med. Sci. Monit..

[11] Mutoh T., Aono H., Seto W., Kimoto T., Tochinai R., Moroi J., Ishikawa T. (2024). Cardiopulmonary Events of the Elderly (≥75 Years) during Clazosentan Therapy after Subarachnoid Hemorrhage: A Retrospective Study from a Tertiary Stroke Center in Japan. Medicina.

[12] Kinoshita H., Kato K., Yamazaki Y., Hashiba E., Hirota K. (2024). Successful Fluid Management in Respiratory Failure due to Clazosentan Following a Cerebral Aneurysm Clipping: A Case Report. Cureus.

[13] Muraoka S., Asai T., Fukui T., Ota S., Shimato S., Koketsu N., Nishizawa T., Araki Y., Saito R. (2023). Real-world data of clazosentan in combination therapy for aneurysmal subarachnoid hemorrhage: A multicenter retrospective cohort study. Neurosurg. Rev..

[14] Mochizuki T., Ryu B., Shima S., Kamijyo E., Ito K., Ando T., Kushi K., Sato S., Inoue T., Kawashima A. (2024). Comparison of efficacy between clazosentan and fasudil hydrochloride-based management of vasospasm after subarachnoid hemorrhage focusing on older and WFNS grade V patients: A single-center experience in Japan. Neurosurg. Rev..

[15] Maeda T., Okawara M., Osakabe M., Yamaguchi H., Maeda T., Kurita H. (2024). Initial real-world experience of clazosentan for subarachnoid hemorrhage in Japan. World Neurosurg. X.

[16] Park J.S., Kang H.G. (2023). Hounsfield unit as a predictor of symptomatic vasospasm and hydrocephalus in good-grade subarachnoid hemorrhage treated with endovascular coiling. Quant. Imaging Med. Surg..

[17] Riley D.S., Barber M.S., Kienle G.S., Aronson J.K., von Schoen-Angerer T., Tugwell P., Kiene H., Helfand M., Altman D.G., Sox H. (2017). CARE guidelines for case reports: Explanation and elaboration document. J. Clin. Epidemiol..

[18] Han J., Lim D.J., Ha S.K., Choi J.I., Jin S.W., Kim S.H. (2016). Endovascular Treatment of Symptomatic Vertebral Artery Dissecting Aneurysms. J. Cerebrovasc. Endovasc. Neurosurg..

[19] Mutoh T., Kazumata K., Terasaka S., Taki Y., Suzuki A., Ishikawa T. (2014). Early intensive versus minimally invasive approach to postoperative hemodynamic management after subarachnoid hemorrhage. Stroke.

[20] Mayer S.A., Aldrich E.F., Bruder N., Hmissi A., Macdonald R.L., Viarasilpa T., Marr A., Roux S., Higashida R.T. (2019). Thick and diffuse subarachnoid blood as a treatment effect modifier of clazosentan after subarachnoid hemorrhage. Stroke.

[21] Higashida R.T., Bruder N., Gupta R., Guzman R., Hmissi A., Marr A., Mayer S.A., Roux S., Weidauer S., Aldrich E.F. (2019). Reversal of Vasospasm with Clazosentan After Aneurysmal Subarachnoid Hemorrhage: A Pilot Study. World Neurosurg..

[22] Thilak S., Brown P., Whitehouse T., Gautam N., Lawrence E., Ahmed Z., Veenith T. (2024). Diagnosis and management of subarachnoid haemorrhage. Nat. Commun..

[23] Mayer S.A., Bruder N., Citerio G., Defreyne L., Dubois C., Gupta R., Higashida R., Marr A., Nguyen T.N., Roux S. (2024). REACT: A randomized trial to assess the efficacy and safety of clazosentan for preventing clinical deterioration due to delayed cerebral ischemia after aneurysmal subarachnoid hemorrhage. J. Neurosurg..

